# BR55 Ultrasound Molecular Imaging of Clear Cell Renal Cell Carcinoma Reflects Tumor Vascular Expression of VEGFR-2 in a Patient-Derived Xenograft Model

**DOI:** 10.3390/ijms242216211

**Published:** 2023-11-11

**Authors:** Jean Courcier, Ingrid Leguerney, Baya Benatsou, Sibylle Pochon, Isabelle Tardy, Laurence Albiges, Paul-Henry Cournède, Alexandre De La Taille, Nathalie Lassau, Alexandre Ingels

**Affiliations:** 1 Department of Urology, Henri Mondor Hospital, University of Paris Est Créteil (UPEC), 94000 Créteil, France; 2 Biomaps, UMR1281, INSERM, Centre National de la Recherche Scientifique (CNRS), Commissariat à l’Energie Atomique (CEA), Université Paris Saclay, 94800 Villejuif, France; 3 Department of Imaging, Gustave Roussy Cancer Campus, 94800 Villejuif, France; 4 Bracco Suisse SA, 1201 Geneva, Switzerland; 5 Department of Urological Oncology, Gustave Roussy Cancer Campus, 94805 Villejuif, France; 6 Laboratory of Mathematics and Computer Science (MICS), CentraleSupélec, Université Paris-Saclay, 91190 Gif-Sur-Yvette, France

**Keywords:** molecular imaging, ultrasound, renal cell carcinomas, VEGFR-2, BR55, patient’s derived xenograft, axitinib

## Abstract

Standard imaging cannot reliably predict the nature of renal tumors. Among malignant renal tumors, clear cell renal cell carcinoma (ccRCC) is the most common histological subtype, in which the vascular endothelial growth factor 2 (VEGFR-2) is highly expressed in the vascular endothelium. BR55, a contrast agent for ultrasound imaging, consists of gas-core lipid microbubbles that specifically target and bind to the extracellular portion of the VEGFR-2. The specific information provided by ultrasound molecular imaging (USMI) using BR55 was compared with the vascular tumor expression of the VEGFR-2 by immunohistochemical (IHC) staining in a preclinical model of ccRCC. Patients’ ccRCCs were orthotopically grafted onto Nod-Scid-Gamma (NSG) mice to generate patient-derived xenografts (PdX). Mice were divided into four groups to receive either vehicle or axitinib an amount of 2, 7.5 or 15 mg/kg twice daily. Perfusion parameters and the BR55 ultrasound contrast signal on PdX renal tumors were analyzed at D0, D1, D3, D7 and D11, and compared with IHC staining for the VEGFR-2 and CD34. Significant Pearson correlation coefficients were observed between the area under the curve (AUC) and the CD34 (0.84, *p* < 10^−4^), and between the VEGFR-2-specific signal obtained by USMI and IHC (0.72, *p* < 10^−4^). USMI with BR55 could provide instant, quantitative information on tumor VEGFR-2 expression to characterize renal masses non-invasively.

## 1. Introduction

Renal Cell Carcinoma (RCC) accounts for 2–3% of all cancers [1]. Its incidence has increased in recent decades, due to incidental detection by ultrasound (US) or computed tomography (CT) scans routinely performed to explore abdominal or back pain [2,3]. In 85% of cases, renal tumors turn out to be malignant, with clear-cell renal cell carcinoma (ccRCC) being the most frequent histological type (75%), followed by papillary renal cell carcinoma (15%), chromophobe renal cell carcinoma (5%) and a multitude of marginal histological types [4]. Small renal masses are distinguished from larger tumors by a higher rate of benign tumors, depending on histology, but also by a lower recurrence probability ratio after treatment [5,6,7]. Benign renal tumors are mainly represented by angiomyolipomas and oncocytomas [4]. Advances in imaging have enabled the better characterization of renal masses [8]. However, it is still impossible to reliably predict the nature of a small renal tumor based on imaging data.

The increasing incidence of small renal tumors calls for new markers to differentiate benign from malignant tumors, and to assess the aggressiveness of malignant tumors to avoid overtreatment of harmless lesions. At present, this problem can partly be solved by biopsies, but this strategy is invasive, sometimes non-contributory and not very effective in predicting the grade of the entire tumor [9]. Developing a method to characterize small renal tumors is, therefore, an urgent medical need.

The radiological diagnosis of small renal masses is commonly based on contrast-enhanced CT, sometimes supplemented by magnetic resonance imaging (MRI) and/or a contrast-enhanced ultrasound [2]. Ultrasound imaging is an easy-to-use, inexpensive, portable and fast technique, with no ionizing radiation and with the ability to assess tumors in real-time. The contrast-enhanced ultrasound is currently receiving particular attention for this purpose. This technique relies on the use of gaseous microbubbles as a contrast agent, which remains in the vascular compartment, providing information on tissues’ vascularization. The dynamic contrast-enhanced ultrasound (DCE-US) enables the quantitative assessment of solid tumor perfusion, using a mathematical model to analyze the raw data [10]. New methodologies, using raw linear data, have been developed to produce increasingly robust semi-quantitative indices of tissue perfusion. Several clinical studies using DCE-US, involving RCC patients, have been proposed to characterize tumors and measure early responses to therapies [11,12].

The development of the ultrasound contrast agents targeted with specific binding ligands to selected molecules expressed on tumor blood vessels has enabled the ultrasound to be used as a molecular imaging technique, allowing the detection of molecular markers overexpressed in tumor tissues [13,14,15]. Ultrasound Molecular Imaging (USMI) uses these targeted microbubbles with binding molecules (peptides, antibodies) presented on their surface [16,17,18]. These targeted microbubbles can bind to biomarkers on the surface of the vascular endothelium, thus enhancing the specific ultrasound signal and enabling the quantification of molecular expression [19,20,21,22].

During tumor growth, the vascular endothelial growth factor (VEGF) and its high-affinity receptor VEGFR-2 are crucial for the formation of new tumor vessels. The VEGFR-2 is known to play a major role in angiogenesis, and particularly in the pathophysiology of renal cell carcinoma, for which drugs targeting the VEGFR-2 pathway are part of the first-line of treatment of metastatic ccRCC, combined with immune checkpoint inhibitors (ICI) targeting the programmed-death pathway (PD-1/PD-L1) [23,24,25].

The VEGF–VEGFR-2 axis, in addition to promoting angiogenesis, could also be involved in stimulating the growth of tumor cells themselves via an autocrine growth factor loop [26]. The VEGFR-2 is overexpressed in ccRCC as a result of tumor neo angiogenesis [27], and it is of particular interest to assess its expression using BR55 (Bracco Suisse SA, Geneva, Switzerland), a VEGFR-2 specific ultrasound contrast agent that has shown promising results on various tumor models [28,29] and is currently the only targeted contrast agent under clinical evaluation for USMI. To date, early clinical trials have been successfully carried out in women with breast and ovarian cancer, showing a strong correlation between VEGFR-2 expression measured by USMI and immunochemistry (IHC) [30] and in men with prostate cancer [31]. Other preclinical studies on BR55 have shown interesting results in preclinical studies [28,32,33,34], but to our knowledge, no application has been explored in renal tumors using USMI with BR55.

These experiments were conducted to explore the enhancement parameters of renal masses during the dynamic phase and the molecular phase of BR55 using USMI: the tumor wash-in, peak intensity, area under the curve, remanent late signal and specific signal from BR55 microbubbles bound to angiogenic vessels in tumor masses.

Afterwards, our main objective was to estimate the correlation between the specific signal of VEGFR-2-targeted microbubbles in tumor masses and the vascular expression of the VEGFR-2 determined by IHC staining in a PdX model of ccRCC.

## 2. Results

### 2.1. Imaging Parameters according to Treatments: Response to Axitinib

Of the 51 mice transplanted, 36 mice were included in the molecular imaging protocol, giving a graft take rate of around 70.6% (the remaining mice showed no or insufficient tumor growth). Graft uptake and tumor growth were monitored by ultrasound until the tumors had reached a minimum volume of 100 mm^3^. Mice were then randomized to vehicle or axitinib-treated groups. All groups combined, the median volume at D0 was 136.8 mm^3^ (interquartile [122.5–164.5 mm^3^]).

**Figure 1:**
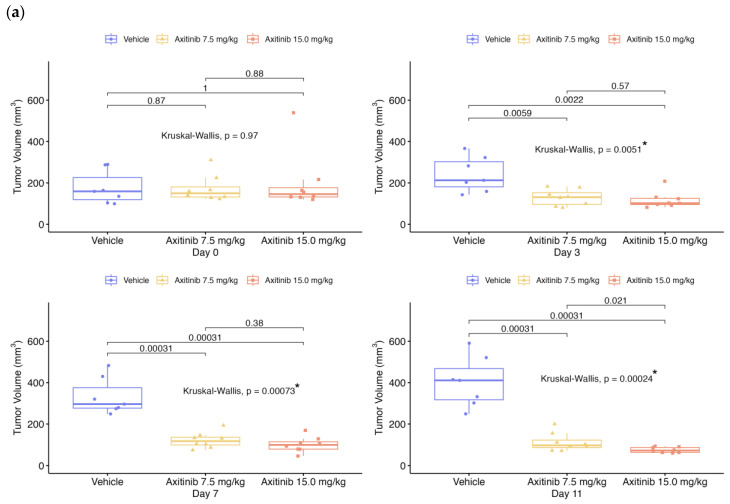

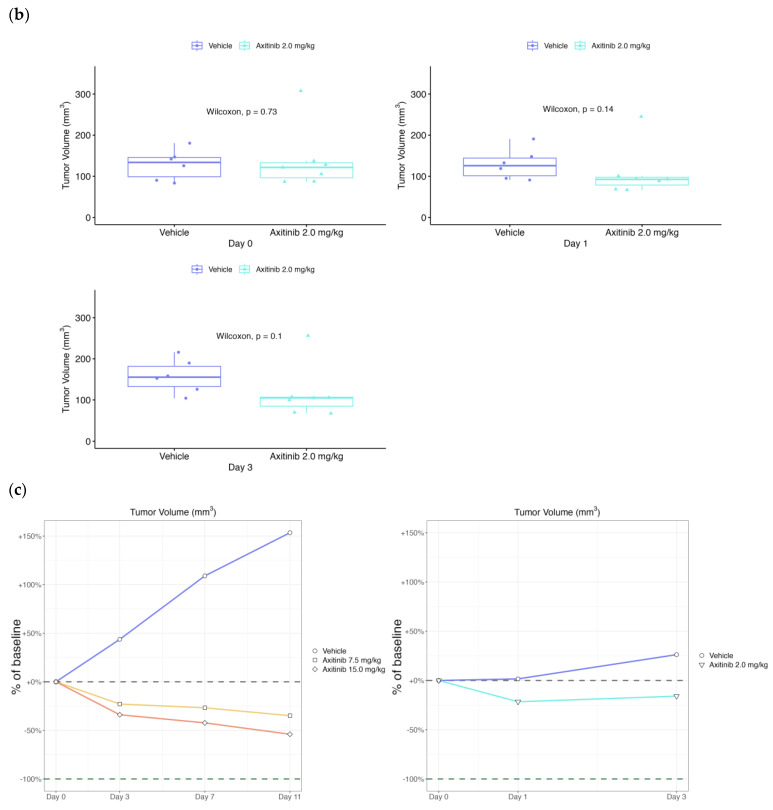
Tumor volume over time for both series of experiments. Data presented for each day and each group with boxplots and individual values: (**a**) in 1st RCC series; (**b**) in 2nd RCC series; (**c**) evolution of the mean according to each group, compared in % from the baseline. The box plots show the median and the interquartile range. Comparisons between two groups are made using the Wilcoxon test. Comparisons between more than two groups are made using the Kruskal–Wallis test. The *p*-value is indicated for the Kruskal–Wallis or Wilcoxon test between vehicle and treated groups, with “*” for significant values.

**Figure 2:**
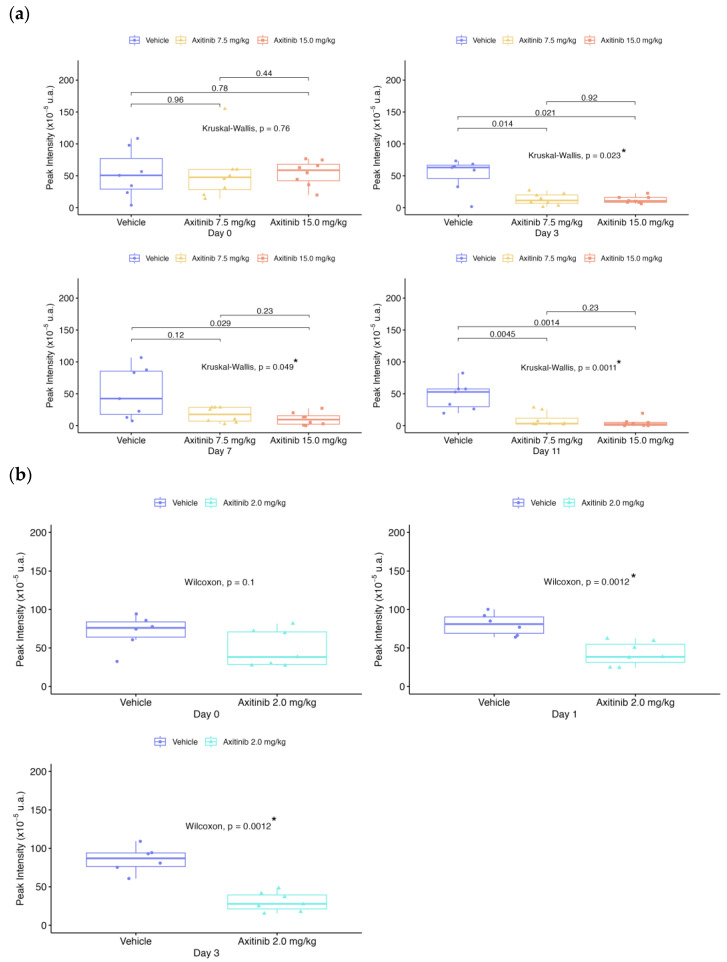

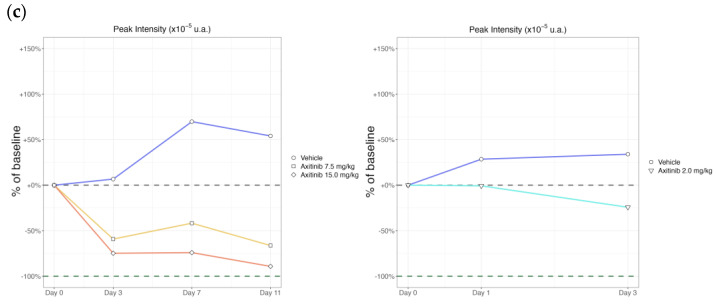
Peak Intensity (PI) over time for both series of experiments. Data presented for each day and each group with boxplots and individual values: (**a**) in 1st RCC series; (**b**) in 2nd RCC series; (**c**) evolution of the mean according to each group, compared in % from the baseline. The box plots show the median and the interquartile range. Comparisons between two groups are made using the Wilcoxon test. Comparisons between more than two groups are made using the Kruskal–Wallis test. The *p*-value is indicated for the Kruskal–Wallis or Wilcoxon test between vehicle and treated groups, with “*” for significant values.

**Figure 3:**
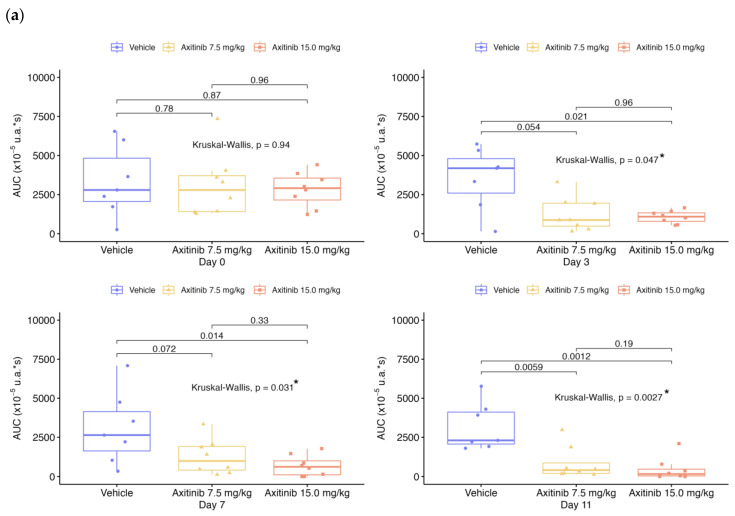

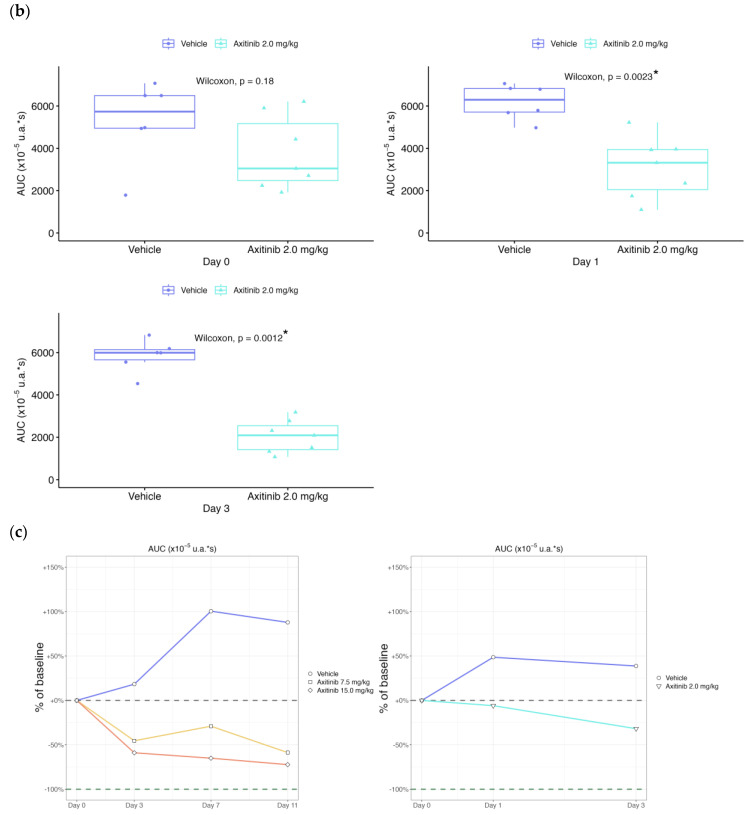
Area under the curve (AUC) over time for both series of experiments. Data presented for each day and each group with boxplots and individual values: (**a**) in 1st RCC series; (**b**) in 2nd RCC series; (**c**) evolution of the mean according to each group, compared in % from the baseline. The box plots show the median and the interquartile range. Comparisons between two groups are made using the Wilcoxon test. Comparisons between more than two groups are made using the Kruskal–Wallis test. The *p*-value is indicated for the Kruskal–Wallis or Wilcoxon test between vehicle and treated groups, with “*” for significant values.

**Figure 4:**
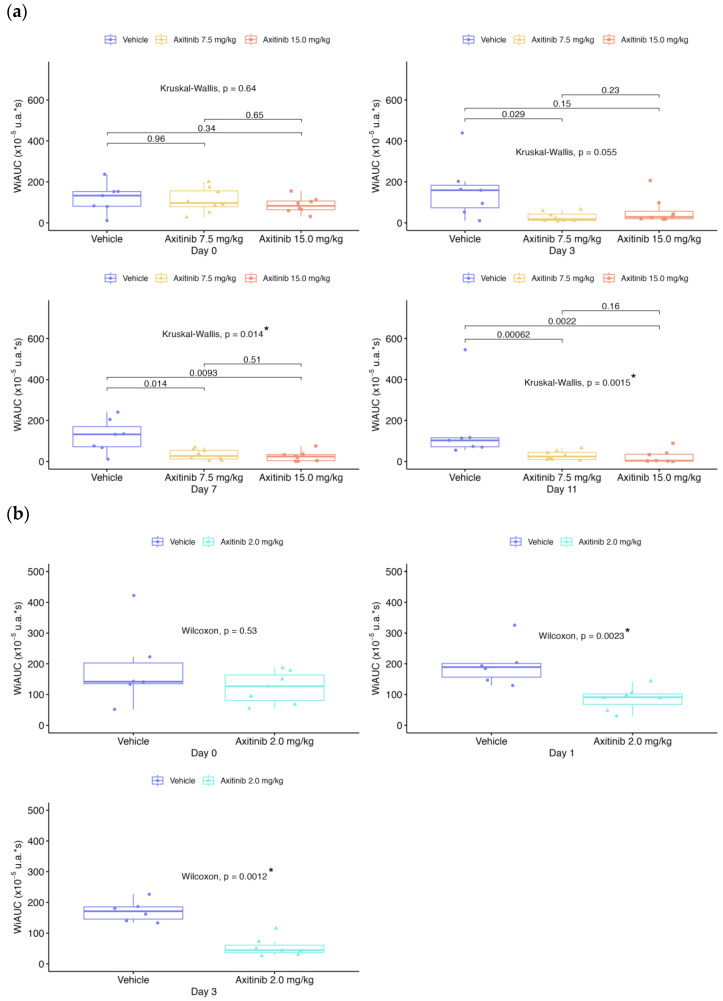

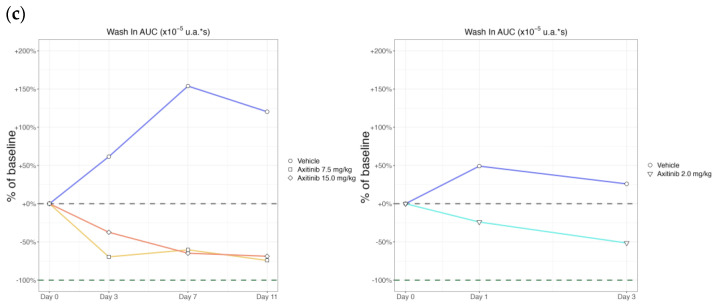
Wash-in Area Under the Curve (WiAUC) over time for both series of experiments. Data presented for each day and each group with boxplots and individual values: (**a**) in 1st RCC series; (**b**) in 2nd RCC series; (**c**) evolution of the mean according to each group, compared in % from the baseline. The box plots show the median and the interquartile range. Comparisons between two groups are made using the Wilcoxon test. Comparisons between more than two groups are made using the Kruskal–Wallis test. The *p*-value is indicated for the Kruskal–Wallis or Wilcoxon test between vehicle and treated groups, with “*” for significant values.

**Figure 5:**
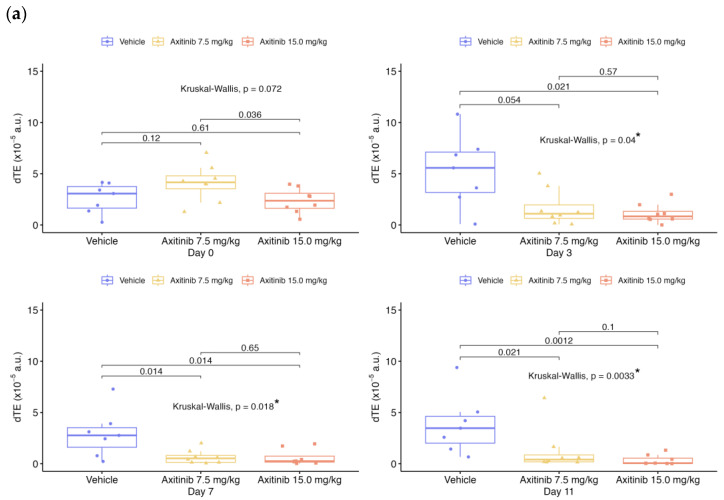

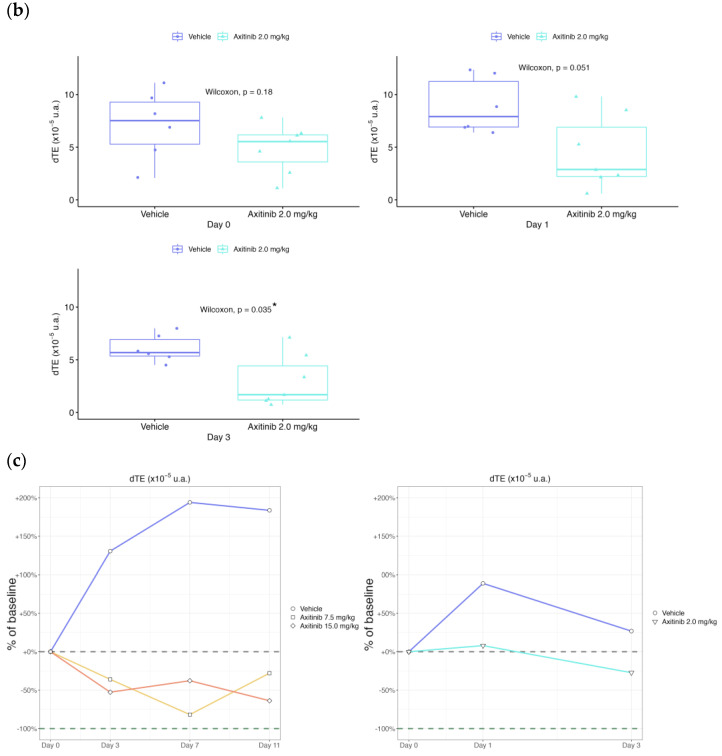
USMI parameter of differential targeted enhancement (dTE) over time for both series of experiments. Data presented for each day and each group with boxplots and individual values: (**a**) in 1st RCC series; (**b**) in 2nd RCC series; (**c**) evolution of the mean according to each group, compared in % from the baseline. The box plots show the median and the interquartile range. Comparisons between two groups are made using the Wilcoxon test. Comparisons between more than two groups are made using the Kruskal–Wallis test. The *p*-value is indicated for the Kruskal–Wallis or Wilcoxon test between vehicle and treated groups, with “*” for significant values.

The tumor volume, perfusion and molecular imaging parameters (PI, AUC, WiAUC, and dTE) for each mouse were assessed on days D0, D1, D3, D7 and D11, depending on the treatment series (Table 1). For Figure 1, Figure 2, Figure 3, Figure 4 and Figure 5, data were presented for each day and each group with box plots and individual values (Figure 1, Figure 2, Figure 3, Figure 4 and Figure 5a,b). In addition, the values of each parameter for each mouse were normalized to the initial value at D0 (Figure 1, Figure 2, Figure 3, Figure 4 and Figure 5c).

On D0, before the start of administration, tumor volumes (Figure 1) were similar between the vehicle and axitinib groups (*p* = 0.97 and *p* = 0.73, according to treatment series). Tumor volumes in axitinib-treated mice were significantly reduced from vehicle groups for doses 7.5 and 15 mg/kg (*p* < 0.001 at D11), but not for dose 2 mg/kg (*p* = 0.10 at D3).

Before treatment (D0), tumor perfusion parameters (Figure 2, Figure 3 and Figure 4) were not significantly different between the vehicle and axitinib groups for PI, WiAUC and AUC. The mean PI and AUC for the axitinib-treated groups decreased significantly, compared with the vehicle group from D3 for the 7.5 and 15 mg/kg doses (*p* < 0.05) and differed from D1 for the 2 mg/kg dose (*p* < 0.005). At D11, the mean PI increased by 54% for the vehicle group and decreased for the axitinib 7.5 and 15 mg/kg groups, with −66.3% and −89.1%, respectively, compared with the baseline (Figure 2). At D3, the mean PI in the axitinib 2 mg/kg group decreased by 24.2%, while the mean PI in the placebo group increased by 34%, compared with D0. The mean AUC (Figure 3) decreased on D3 for the axitinib 2 mg/kg group (−31.8%), compared with the vehicle group (+38.7%), and decreased at D11 by −58.7% and −72.3% for the axitinib groups (doses 7.5 and 15 mg/kg, respectively), in contrast with the vehicle group (+87.8%). WiAUC data were similar to those obtained for AUC for the axitinib groups compared with the baseline and vehicle values (Figure 4).

The VEGFR-2-specific signal assessed in the late phase by the dTE parameter (Figure 5) decreased significantly in the axitinib 2 mg/kg group at D3 with −27.3%, and at D11 in the 7.5 and 15 mg/kg groups with −27.8% and −67.8%, respectively. The dTE after the baseline was significantly different for the axitinib and vehicle groups on each day (*p* < 0.05), except at D1 in the axitinib 2 mg/kg group.

Statistical results between vehicle and treated groups for all parameters shown in Figure 1, Figure 2, Figure 3, Figure 4 and Figure 5 are summarized in Table 2.

### 2.2. Comparison of USMI and IHC Measurements

Tumor sections analyzed by IHC were compared with the ROI chosen for the USMI parameter analysis, to ensure that IHC measurements were made on a section very close to, or identical with, the section chosen for USMI measurements. Ultrasound size measurements were slightly higher than those obtained on histology sections, certainly linked to the resolution of each imaging technique, and to tissue shrinkage during fixation. A strong correlation confirmed the correspondence between surfaces assessed by ultrasound and by pathology (Pearson correlation ρ = 0.84 (95%CI [0.71–0.92], *p* < 10^−4^).

**Figure 6:**
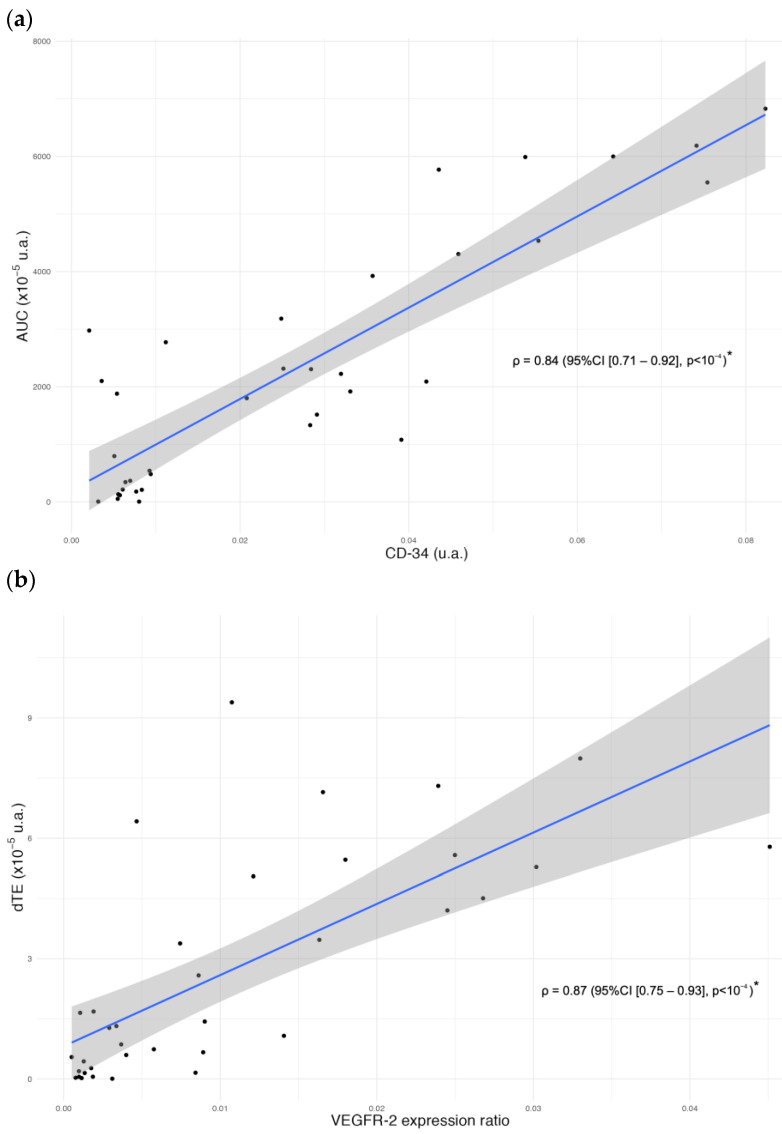
(**a**) Correlation (ρ = 0.87, *p* < 10^−4^) between AUC and CD34 expression relative to total surface area in IHC and (**b**) Correlation (ρ = 0.72, *p* < 10^−4^) between bound MBs signal intensity measured on late-phase BR55 ultrasound and area of VEGFR-2 expression in IHC, relative to tumor surface area. The outer edges of the shaded area represent the confidence bands, indicating the 95% confidence intervals for the mean of the Y-variable at each value of the X-variable. The *p*-value is indicated with “*” for significant values.

Functional parameters of the tumor perfusion curves were correlated with CD34 immunostaining. AUC was significantly correlated with the CD34 ratio for each data set, with a Pearson correlation of ρ = 0.87 (95%CI [0.75–0.93], *p* < 10^−4^), including all measurements (Figure 6a).

The specific dTE signal was significantly correlated with VEGFR-2 immunostaining (Figure 6b), confirming its expression in endothelial cells, with a Pearson correlation of ρ = 0.72 (95%CI [0.51–0.84], *p* < 10^−4^).

## 3. Discussion

Our team performed a preclinical study on a ccRCC PdX model. The human ccRCC was implanted under sterile conditions in the renal subcapsular position in 36 immunodeficient mice. Imaging sessions were performed on mice divided into groups treated with the placebo or axitinib. The tumor volume and perfusion parameters were assessed following the intravenous injection of BR55.

In this study, we observed a linear correlation (ρ = 0.72 (95%CI [0.51–0.84]), *p* < 10^−4^) between specific BR55 enhancements by the ultrasound and the VEGFR-2 expression measured by IHC in ccRCC tumors.

The dose of axitinib treatment was chosen cautiously after a review of the literature, in order to nuance the anti-tumor activity without serious adverse treatment effects [35]. The design of the experiment with subjects divided into four groups had the major objective of maximizing inter-individual tumor heterogeneity in terms of size, vessel density and intra-tumoral VEGFR-2 expression. Axitinib is a well-described inhibitor of the VEGFR-2 pathway used in clinical practice in advanced ccRCC [23]. A decrease in tumor expression of the VEGFR-2 has been described in ccRCC in preclinical models as a consequence of the axitinib effect [36]. Thus, the expected effect of axitinib on PdX tumors was to repress vascular VEGFR-2 expression in a dose-dependent manner.

Upon close examination of the correlations between USMI and IHC, a significant number of spots were exhibited, which showed a low BR55 intensity and weak VEGFR-2 IHC staining. These points corresponded to mice treated with the highest doses of axitinib (7.5 mg/kg and 15 mg/kg). This finding implies that the efficacy of theses doses of axitinib may have exceeded the desired level, compared to the lower doses. This hypothesis is supported by the observation of significant tumor shrinkage in the treated groups compared with the placebo, right from the first ultrasound evaluation. Moreover, the intrinsic sensitivity of the tumor response to anti-angiogenic treatments was not predictable, and we hypothesize that the model used here with these human ccRCCs was particularly sensitive to axitinib, probably as a function of the histo-pathological particularities of these RCCs and the characteristics of the parental tumors [37].

Another notable difficulty in carrying out the protocol was to ensure that the tumor surfaces measured by BR55 USMI corresponded to the surfaces analyzed by IHC. To this end, the careful identification of the area of interest was carried out prior to the BR55 USMI measurements, and the tumor was cut in the same plane before the paraffin embedding. The comparison between the area of interest estimated in the BR55 USMI and the digitized tumor surface measurement showed an excellent correlation ρ = 0.84 (95%CI [0.71–0.92], *p* < 10^−4^).

We chose to constitute our mouse cohorts with a second-pass xenograft to ensure a certain capacity for tumor growth with an equivalent growth rate between groups [38]. Nevertheless, tumor heterogeneity is known to be constitutive of ccRCC [39,40] and we were unable to ensure perfect tumor homogeneity between PdX subjects, compared with the use of well-established tumor cell lines. On the other hand, it is more likely that this model based on real patient tumors therefore reproduces a “close to reality” tumor complexity with its microenvironment and its parental peritumoral vasculature [41,42].

Looking for a non-invasive method to characterize the malignant or benign nature of small renal masses (<4 cm), the VEGFR-2 seems to be a relevant biomarker to be explored by USMI with BR55. Our central hypothesis is that small renal malignancies may exhibit a distinct pattern of VEGFR-2 expression, which differs from that observed in benign or indolent tumors. A contrast ultrasound based on the injection of contrast agents has shown its potential interest in the active follow-up of small renal tumors [43,44]. Small renal tumors would be an ideal field of application for the BR55 molecular ultrasound technique, since clear cell renal cell carcinoma represents the vast majority of renal carcinomas and shows the hyperactivation of neoangiogenesis and of the hypoxia-inducible factor/vascular endothelial growth factor (HIF-VEGF) pathway [45]. VEGF expression has been described as a marker of tumor aggressiveness [46,47]. In addition, their small size means that the entire tumor volume can be analyzed, providing global information on VEGFR-2 expression within the tumor.

To our knowledge, an assessment of VEGFR-2 expression in IHC according to histological subtypes in small renal masses has not been described, although authors have reported higher VEGF or VEGFR-2 expression in papillary RCC, compared with ccRCC [47]. The potential application of BR55 by USMI would lie in the ability to discriminate small malignancies from benign renal masses, and we assume that it should constitute exploratory research.

To date, early clinical trials have been successfully carried out in women with breast and ovarian cancer, showing a strong correlation between VEGFR-2 expression measured by USMI and immunochemistry (IHC) [30] and in men with prostate cancer [31].

The specific BR55 signal measured by USMI correlated strongly (ρ = 0.72 (95%CI [0.51–0.84]), *p* < 10^−4^) with VEGFR-2 expression determined by immunohistochemistry on tumor sections at day 3 and day 11 after the treatment induction, demonstrating the potential of BR55 to characterize human renal tumors that express the VEGFR-2.

## 4. Materials and Methods

### 4.1. Ethics Statement

The free and informed consent of each patient was obtained in writing prior to the procedure for the collection, storage and use of tumor fragments for scientific research purposes. All animal experiments were carried out in agreement with the national ethics committee (CEEA 26, Paris-Sud University) and the French Ministry of Agriculture (approval number: APAFIS#8963-2017022014433962). The animals were raised and housed in the animal facility (Experimental Preclinical Evaluation Platform, PFEP, Gustave Roussy, France) and received all necessary care in accordance with the regulatory recommendations on animal welfare [48].

### 4.2. Renal Cell Carcinoma Tumor Model: Patients’ Characteristics

Clear cell RCCs were harvested from two nephrectomy samples from two patients (1st RCC and 2nd RCC), enabling the development of experimental cohorts. A total of 36 mice were transplanted and included in the protocol. These were selected for their similar growth rate, enabling us to compare data from the two series, while having a panel representative of tumor heterogeneity. Tumors were randomized to vehicle or axitinib. The animal cohorts are shown in Table 1.

### 4.3. Orthotopic Transplants of Human RCC

Initially, the kidney was removed after the patient underwent radical nephrectomy for renal cancer. Once extracted, the surgical specimen was preserved in a cold tissue preservative solution at 4 °C (Custodiol^®^, EUSA PHARMA SAS, Lyon, France), and then an 8 mm diameter core was extracted under sterile conditions from the tumor area of interest. The tumor sample was chosen in agreement with the pathologist, in a non-cystic, non-necrotic area that would not interfere with the analysis of the tumor resection margins. The tumor core was then transported, isolated in the same cooled preservation liquid (Custodiol^®^), to the animal experimentation laboratory. Tumor slices, 300 μm thick, were cut using a microtome (Krumdiek tissue slicer) and then grafted into a renal subcapsule in mice, under sterile conditions, as described in previous studies [22,49,50].

The animals used were highly immunocompromised Nod-Scid-Gamma (NSG) or Nod-Scid-IL2rγnull (NSG-IL) mice. Mice were anesthetized with a mixture of 2% isoflurane and air insufflated at 2L/min. Anesthesia was then maintained at the mask by means of an adapted device, coupled with a heating plate and a ventilation–aspiration mouth for the anesthetic gas mixture. The modulation of the isoflurane level was sometimes necessary to adapt to the animal’s clinical tolerance. The animals were placed in the left lateral decubitus position, to expose their entire right flank. Before the surgical implantation of the fragment under the renal capsule, the animal’s flank was shaved and cleansed with 70% medical alcohol and 10% povidone iodine for dermal use.

### 4.4. Experimental Groups

The engraftment growth in the kidney was monitored by the B-mode ultrasound and Doppler imaging until a tumor with a minimum volume of at least 100 mm^3^ was obtained. Mice were then divided into groups receiving either the vehicle solution or axitinib (Pfizer, New York, NY, USA). Axitinib is indicated as a first-line treatment for metastatic renal cell cancer (mRCC) in combination with the anti-PD1 immune checkpoint inhibitor (Pembrolizumab) [51]. Axitinib is a tyrosine kinase inhibitor, which specifically blocks the signaling cascade, and therefore, the biological activity of the vascular endothelial growth factor (VEGF) at its receptors (VEGFR-1, VEGFR-2, VEGFR-3).

The vehicle solution (methocel 0.5%) and axitinib treatments were administered by oral gavage, at a dose of 10 mL/kg per day (vehicle), and 2, 7.5 and 15 mg/kg (axitinib treatment).

A first series of evaluations with the RCC model was carried out with vehicle versus axitinib 7.5 and 15 mg/kg groups at times D0, D3, D7 and D11, D0 being the date of the allocation of mice within groups, the baseline USMI imaging and followed by the first administration of treatment. A second series of evaluations was carried out in the RCC model comprising a vehicle versus axitinib 2 mg/kg group at D0, D1 and D3 (Table 1).

### 4.5. Ultrasound Molecular Imaging

All ultrasound imaging sessions were performed using an APLIO500 ultrasound scanner (Canon Medical System, Otawara, Japan), equipped with a 12 MHz linear probe (PLT-1204BT). Each mouse was imaged under gas anesthesia (2% isoflurane in air at 1.5 L/min), and were maintained on a thermostatically-heated table throughout the examination. The tumor dimensions were measured along the longest axes using B-mode imaging to estimate the tumor volume in cubic millimeters. Tumor perfusion and angiogenesis were then assessed by injecting BR55, a specific contrast agent targeting the VEGFR-2 (Bracco Suisse SA, Geneva, Switzerland) [52]. It comes in the form of a lyophilizate containing 2.5 × 10^9^ microbubbles (MBs) after a reconstitution with 2 mL of 5% glucose. The microbubbles have an average diameter of 1.7 µm and consist of a lipid membrane containing a gaseous body composed of a mixture of perfluorobutane (chemical formula C4F10) and nitrogen (N2). The outer surface of the microbubbles’ membrane is decorated with heterodimeric peptides, having a high affinity for the VEGFR-2 [28].

**Figure 7:**
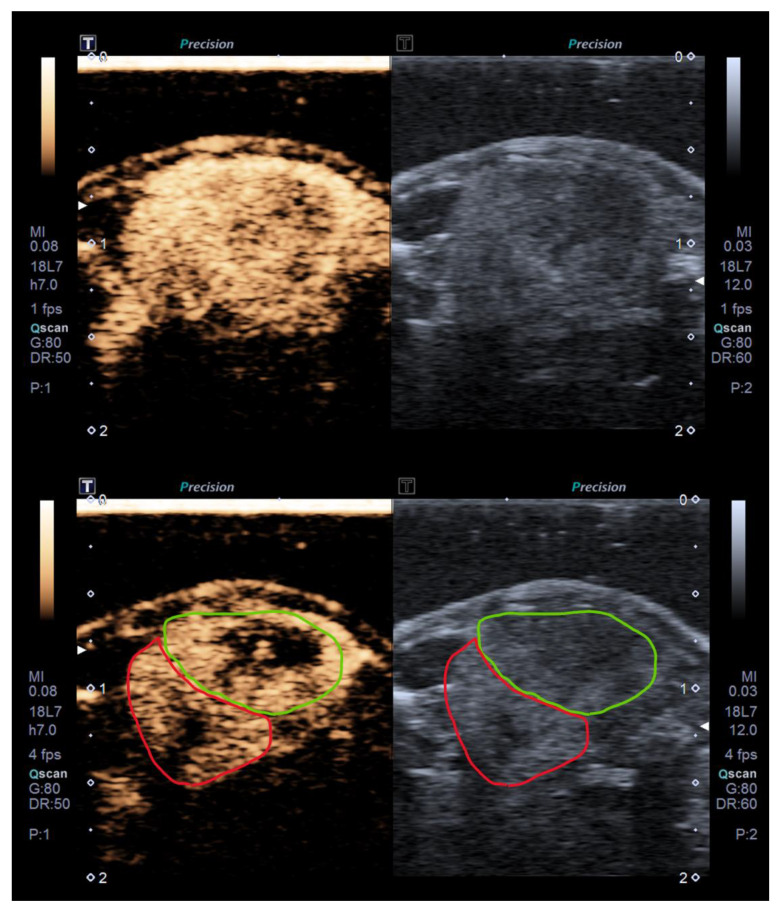
BR55 US images at maximum peak intensity in dynamic phase (**top**) and at late phase (**bottom**), in DUAL mode, enabling the tumor to be visualized simultaneously during the same acquisition in B mode (**right**) and contrast mode (**left**). Images are acquired in the plane of the longest axis of a PdX orthotopic renal tumor. Kidney and tumor boundaries are indicated in the lower image with red (kidney) and green (tumor) outlines.

Molecular imaging was performed along the longest axis of the tumor, simultaneously displaying the B-mode and pulse-subtraction contrast mode (DUAL display as shown in Figure 7) for the precise delineation of the region of interest (ROI). BR55 was injected at a dose of 3 × 10^8^ MBs/kg at a volume of 50 µL. The recording of contrast imaging sequences began as soon as BR55 was injected, and took place in two phases: an early acquisition phase during BR55 injection (90 s, 7 MHz, MI = 0.08, acquisition at 1 fps) to record the enhancement of the ultrasound signal, followed by the decay phase.

The ultrasound acquisition was then reactivated for the late acquisition phase at 10 min (75 s, 7 MHz, MI = 0.08, 4 fps) followed by a high MI (1.53) destructive ultrasound pulse, enabling the destruction of circulating and targeted microbubbles attached to the neo-vessel endothelium. The difference in the ultrasound signal (differential Targeted Enhancement, dTE) was calculated between the signal before destruction and the signal after destruction, resulting from the replenishment of the tissue with residual circulating microbubbles. Ultrasound imaging sequences were exported in the DICOM format for post-processing and signal quantification. ROIs were delineated by two highly experienced examiners.

The tumor perfusion intensity was quantified in each ROI over time, and the following perfusion parameters were determined from the time-intensity curves: (i) during the dynamic phase, peak intensity (PI), wash-in area under the curve (WiAUC) and area under the curve (AUC) comprising the two areas under the curve during the “wash-in” and “wash-out” phases; (ii) during the late-phase, dTE, as the amount of the VEGFR-2 in the ROI.

USMI ultrasound sessions with BR55 were performed at D0, then at D1, D3, D7 and D11 after the start of the treatment administration. Animals were sacrificed on D3 or D11, depending on the treatment series, to allow tumor harvesting for an immunohistochemistry analysis.

### 4.6. Immunohistochemistry

**Figure 8:**
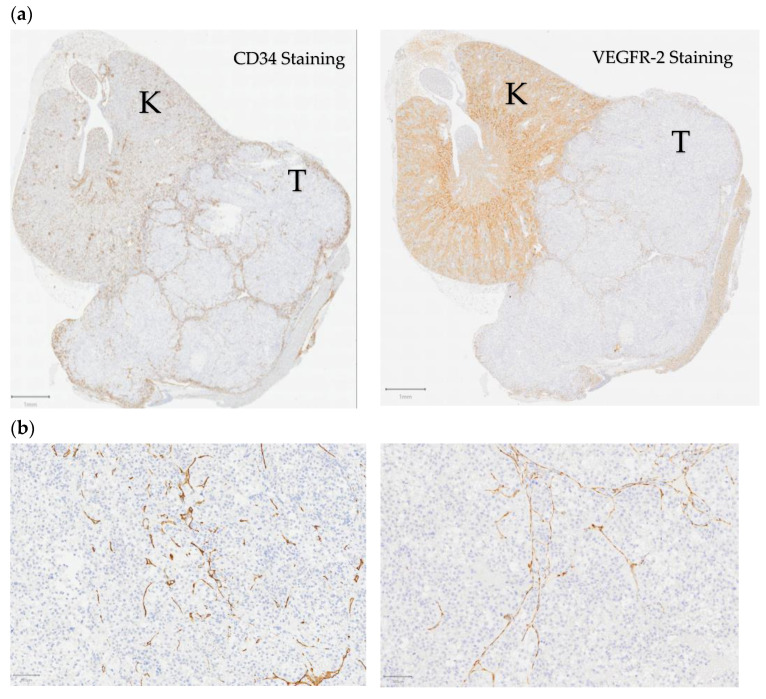
IHC-stained digitized slides of orthotopically PdX-grafted ccRCC tumors. (**a**) Section showing tumor (T) and kidney (K) stained for CD34 (**left**) and VEGFR-2 (**right**), respectively. Bar = 1 mm. (**b**) Zoom images within the tumor; bar = 100 μm.

The plane explored in the USMI was determined for each tumor on day 0 and served as a reference for follow-up by USMI. The tumor removal was performed at the end of the last imaging session. The position of the mouse was maintained during removal, and the tumors were cut in two parts along the axis determined by the respective ultrasound session at D3 and D11. Tumors were then fixed in a 4% paraformaldehyde solution diluted in PBS, embedded in paraffin and cut to a thickness of 4 μm with a microtome (Leica RM2245, Leica Biosystems, Wetzlar, Germany). Staining was performed by the PETRA platform (Experimental and Translational Pathology, Gustave Roussy Cancer Campus, Villejuif, France): standard HES (Hematoxylin Eosin Safran) staining to visualize the tissue integrity and morphology, immunolabeling with rat anti-mouse CD34 antibody (1:20, HM1015, clone MEC14.7, Hycult Biotech, Uden, The Netherlands) and rabbit monoclonal antihuman VEGFR-2 antibody (1:100, #2479, clone 55B11, Cell Signaling Technology, Danvers, MA, USA), and detected by the Bond Polymer refined detection kit (Leïca Biosystems, #DS9390). IHCstained slides were scanned in their entirety at 20× magnification (VS120, Olympus Life Science Solutions, Tokyo, Japan), as shown in Figure 8. Computer processing was performed using the open source software QuPath v0.3.1 for the Quantitative Pathology and Bioimage Analysis [53]. The delineation of each tumor was performed manually to determine its surface area. DAB staining was selected by applying an intensity threshold filter, identical for all slides. The staining percentage for each slide was calculated as the ratio of the number of positive pixels to the total tumor tissue area (positive pixels count), for both CD34 (CD34 ratio) and VEGFR-2 (VEGFR-2 ratio). The threshold applied determined that the positive staining (positive pixels) was the same for all tumors.

### 4.7. Statistical Analysis

Results are reported as the mean, median and interquartile range (IQR). The tumor response was assessed by normalizing each parameter to its pre-treatment value. Comparisons of quantitative variables between the two groups were made using a Wilcoxon test, and those within the same group measured at different time points using a Wilcoxon test for paired data. Inter-group comparisons of quantitative variables were made using the non-parametric Kruskal–Wallis test. Comparisons between two variables were assessed using Pearson’s correlation test. The significance threshold was set at *p* < 0.05. All statistical analyses were performed using the R Project for Statistical Computing [54].

## Figures and Tables

**Table 1:** Experimental design of the protocol.

	Days of Evaluation	D0	D1	D3	D7	D11
1st RCC	Vehicle (n = 7)	USMI ^1^		USMI	USMI	USMI + IHC ^2^
	Axitinib 7.5 mg/kg (n = 8)	USMI		USMI	USMI	USMI + IHC
	Axitinib 15 mg/kg (n = 8)	USMI		USMI	USMI	USMI + IHC
2nd RCC	Vehicle (n = 6)	USMI	USMI	USMI + IHC		
	Axitinib 2 mg/kg (n = 7)	USMI	USMI	USMI + IHC		

^1^ USMI: UltraSound Molecular Imaging. ^2^ IHC: ImmunoHistoChemistry.

**Table 2:** Statistical results per day for all parameters assessed for the two series. The *p*-value is indicated for the Kruskal–Wallis or Wilcoxon test between vehicle and treated groups, with “*” for significant values.

	Days of Evaluation	Tumor Volume	PI	AUC	WiAUC	dTE
1st RCC	D0	*p* = 0.97	*p* = 0.76	*p* = 0.94	*p* = 0.64	*p* = 0.072
	D3	*p* = 0.0051 *	*p* = 0.023 *	*p* = 0.047 *	*p* = 0.055	*p* = 0.04 *
	D7	*p* = 0.00073 *	*p* = 0.049 *	*p* = 0.031 *	*p* = 0.014 *	*p* = 0.018 *
	D11	*p* = 0.00024 *	*p* = 0.0011 *	*p* = 0.0027 *	*p* = 0.0015 *	*p* = 0.0033 *
2nd RCC	D0	*p* = 0.73	*p* = 0.1	*p* = 0.18	*p* = 0.53	*p* = 0.18
	D1	*p* = 0.14	*p* = 0.0012 *	*p* = 0.0023 *	*p* = 0.0023 *	*p* = 0.051
	D3	*p* = 0.1	*p* = 0.0012 *	*p* = 0.0012 *	*p* = 0.0012 *	*p* = 0.035 *

## Data Availability

The data is available on request from the corresponding authors.
